# Induction Phase of Spontaneous Liver Transplant Tolerance

**DOI:** 10.3389/fimmu.2020.01908

**Published:** 2020-09-11

**Authors:** Geoffrey W. McCaughan, David G. Bowen, Patrick J. Bertolino

**Affiliations:** ^1^Liver Injury and Cancer Program, The Centenary Institute, University of Sydney and Royal Prince Alfred Hospital, Sydney, NSW, Australia; ^2^AW Morrow Gastroenterology and Liver Centre, University of Sydney and Royal Prince Alfred Hospital, Sydney, NSW, Australia; ^3^Liver Immunology Program, The Centenary Institute, University of Sydney and Royal Prince Alfred Hospital, Sydney, NSW, Australia

**Keywords:** Transplantation, liver allograft, tolerance, hepatocytes, passenger leucocytes, suicidal emperipolesis, high antigen load, T cells

## Abstract

The liver has long been known to possess tolerogenic properties. Early experiments in liver transplantation demonstrated that in animal models, hepatic allografts could be accepted across MHC-mismatch without the use of immunosuppression, and that transplantation of livers from the same donor was capable of inducing tolerance to other solid organs that would normally otherwise be rejected. Although this phenomenon is less pronounced in human liver transplantation, lower levels of immunosuppression are nevertheless required for graft acceptance than for other solid organs, and in a minority of individuals immunosuppression can be discontinued in the longer term. The mechanisms underlying this unique hepatic property have not yet been fully delineated, however it is clear that immunological events in the early period post-liver transplant are key to generation of hepatic allograft tolerance. Both the hepatic parenchyma and the large number of donor passenger leukocytes contained within the liver allograft have been demonstrated to contribute to the generation of donor-specific tolerance in the early post-transplant phase. In particular, the unique nature of hepatic-leukocyte interactions appears to play a crucial role in the ability of the liver to silence the recipient alloimmune response. In this review, we will summarize the evidence regarding the potential mechanisms that mediate the critical early phase in the generation of hepatic allograft tolerance.

## The Spontaneous “Liver Tolerance Effect”

It was first recognized over 50 years ago that outbred pigs could spontaneously accept liver allografts indefinitely without any immunosuppressive treatment (1). This finding has since been confirmed in wild type mice (2), as well as in many inbred mouse (3) and rat (4) strains. In a series of elegant experiments in outbred rats, recipients of liver allografts also accepted subsequent skin or heart transplants from the same donor strain while rejecting third party grafts [reviewed in (5)]. These experiments formally demonstrated that the liver, in addition to being spontaneously accepted, can also induce donor strain-specific tolerance to subsequent transplants of other tissues. Furthermore, a liver transplant (LT) is able to reverse severe on-going graft rejection of a previous organ transplant from the same donor strain, including heart, pancreas and skin, thus conferring donor specific immunity.

When liver transplantation became accepted clinical practice in the early 1980s, it was expected that this so-called “liver tolerance effect” would also be seen in humans. Indeed, patients who receive liver allografts do require less immunosuppression than recipients of other organs, and successful weaning off immunosuppressive therapy, either intentional or forced by lympho-proliferative disorders or life-threatening infections, has been reported (6). However, this has been observed in only in a small subset of patients (to be discussed later).

When considering LT tolerance, regardless of whether it is in an experimental or clinical setting, it is worth splitting it conceptually into two distinct periods: induction and maintenance phases. This review will concentrate on the former but will contrast it to the latter.

## The Induction Phase—Early Studies

Following the seminal observation by Sir Roy Calne in 1969 that pig liver allografts are spontaneously accepted (1), most studies in LT have been performed using rat models. LT in rats is technically easier than in mice, as their vessels are 8 times larger and more readily manipulated. This reduces hepatic ischemic time during surgery and increases the success rate of this procedure. In addition, the outcome of rat liver transplantation is genetically determined by the donor and recipient strain: while some strain combinations result in liver allograft acceptance, others lead to rejection. This not only recapitulates the two possible outcomes in clinical LT, but also allows side by side comparisons to be made between accepting and rejecting strain combinations. In their original experiments in rats, Kamada et al. (4) made several important mechanistic discoveries. Firstly, during the first days after LT there was little difference in liver injury between rats that would ultimately accept their liver allograft and recipients who subsequently rejected their liver transplant, as assessed by liver enzyme levels and intrahepatic cellular infiltrates. However, while liver enzyme elevation and cellular infiltrates were transient and returned to normal within a few weeks in tolerant animals, they progressively increased in non-tolerant rats leading ultimately to allograft rejection by day 18 in the absence of immunosuppression. The mechanisms underlying these intriguing findings were further explored in subsequent studies (7) that tracked anti-donor cytotoxic T cells in different compartments of tolerant animals. These studies revealed that the thoracic duct that ultimately drained the tolerant liver was significantly depleted of anti-donor cytotoxic T cell reactivity; rather, this reactivity accumulated in the liver allograft at early time points (7). This paradoxical observation was surprising at the time, as the intrahepatic accumulation of potentially harmful T cells did not lead to graft rejection, but was instead associated with tolerance induction and graft acceptance. The most plausible explanation for these findings was that alloreactive CD8 T cells were retained in the liver, resulting in their systemic depletion in the recipient (see below), and were subsequently silenced *in situ* within the tolerated liver allograft. To determine whether T cells were silenced early by regulatory T cells (termed suppressor T cells at that time), splenic cells harvested from tolerant LT animals within the first 30 days post-transplant were adoptively transferred into recipients of irradiated livers that would usually undergo rejection in the same strain. Such treatment failed to reliably induce tolerance of irradiated liver allografts (8). Whether antibodies mediated LT tolerance in the rat was investigated by serum transfer from tolerant LT recipients, which also failed to induce tolerance in the majority of recipients [reviewed in (5)]. These studies indicated that the T cell silencing mechanisms that regulate spontaneous acceptance of liver allografts were not mediated solely by circulating anti-donor antibodies or regulatory T cells but involved other mechanisms. These findings suggested that these non-regulatory mechanisms occurred at an early time point, within the first few days post-surgery, within the liver allograft itself.

## The Induction Phase—Subsequent Studies in Rats

In some of our early studies, we used a combination of immunohistology and quantitative PCR methods to analyze and compare the intrahepatic responses in tolerant and rejecting animals at days 3–5 post-LT (9). To our surprise, but consistent with the histological and biochemical observations previously made by Kamada and colleagues, tolerant and rejecting livers expressed similar levels of CD4, CD8, CD3 cells and IL-2, interferon-γ, IL-4, and IL-10 mRNA (9). However, our subsequent experiments revealed increased intrahepatic lymphocyte apoptosis in the tolerant liver, suggesting that T cells retained in the liver died or were cleared *in situ* (10). Fas-FasL mediated T cell death of CD8 T cells after rat liver transplantation was also reported by Dresske et al. (11).

At approximately the same time, Starzl et al. provided evidence for early migration of donor, or passenger, lymphocytes (PLs) from the hepatic allograft into systemic lymphoid tissues, and demonstrated that the lymphoid tissues of recipients accepting a liver allograft contained donor cells that survived months after liver transplantation (12). Based on this observation, they suggested that chimerism was the tolerance mechanism driving liver allograft acceptance. They hypothesized that tolerance resulted from an equilibrium between two limited antagonistic graft-versus-host and host-vs.-graft responses that would stabilize over months (12). One of the major criticisms of this model is that it remains unclear whether the survival of donor PL in the recipient is a consequence rather than the cause of tolerance in the recipient. Furthermore, although microchimerism is observed in some LT patients, it does not explain why some LT patients accepted their liver allografts without any sign of microchimerism (13). Despite these concerns, this model has profoundly influenced the LT field by inspiring subsequent studies that investigated the potential key role of PLs in tolerance [reviewed in more detail by (14)].

Although our experiments in rats confirming PL migration within 24 h post-LT, we were unable to identify persistence of donor cells, indicating that they failed to establish microchimerism. Our studies also indicated that the degree of donor cell migration was the same in tolerant vs. rejecting strain combinations (15). Our cytokine studies subsequently revealed a significant but important paradox. Rather than finding increased level of immune activation cytokine mRNA in the lymph nodes and spleen of rejecting animals, our findings revealed the opposite: IL-2 and interferon-γ mRNA expression levels were significantly higher in lymphoid tissues of tolerant vs. rejecting recipients (15). Cytokine levels peaked at 24 h post-transplant (15) and their main source were donor CD4 T cells. Subsequent studies showed that high cytokine levels were associated with increased lymphocyte apoptosis (10). In contrast to IL-2 and interferon-γ, TGF-β, IL-6, TNF-α, and IL-10 mRNA levels were similar between tolerant and rejecting animals (15). Supporting a key role for donor passenger leucocytes in inducing LT tolerance, irradiation of the donor livers before transplantation, which results in depletion of intrahepatic leukocytes, abrogated spontaneous acceptance of the donor liver, resulting in rejection (16). Furthermore, acceptance of irradiated livers was restored when large numbers of donor splenocytes were adoptively transferred into recipients or the irradiated donor liver was “parked” for 24 h allowing re-constitution of the original intrahepatic leucocyte population (16). These findings were notable, as this was the first time that spontaneous LT tolerance in the rat model had been abrogated. By comparing three different rat transplantation models without immunosuppression (small bowel, liver and liver/small bowel transplantation), Meyer et al. (17) showed that donor cell numbers persisting in the spleen 100 days after transplantation were not significantly different during rejection and tolerance. They concluded that “the allograft determines the presence of peripheral donor cells rather than being influenced itself by their existence.” However, the same study demonstrated that tolerance was associated with persistence of donor cells identified as DCs and KCs in the allograft itself. This graft chimerism seems to be unique to the liver and might explain the unique tolerance inducing properties of this organ.

In a seminal study, Calne et al. (18) performed a series of elegant experiments in rats in which they assessed the role of the parenchyma and donor PLs in tolerance induced by liver allografts. To assess the role of donor PL, they performed “parking” experiments in which they transplanted donor livers into allogeneic recipients to reconstitute the donor liver with recipient leucocytes. After 20 days, liver grafts were removed and transplanted into second recipients, thus allowing analysis of the role of donor liver parenchyma vs. PLs in LT tolerance. Recipients of a chimeric liver containing PLs syngeneic with transplanted skin but parenchyma syngeneic with the recipient subsequently rejected skin grafts (18), suggesting that expression of donor MHC restricted to donor PLs was not sufficient to induce tolerance, and that the liver parenchyma itself was necessary for the induction of spontaneous LT tolerance. Similar findings were obtained in other studies (11, 19, 20). By generating bone marrow radiation chimeras in which the liver contains hematopoietic antigen-presenting cells of a different genotype than the parenchymal tissue, Kreisel et al. showed that although PL influenced the tempo of rat liver graft rejection and were important for inducing liver tolerance, this immunological unresponsiveness was not dependent on the presence of antigen-presenting cells of donor type (20).

The findings by Kamada et al. that immune infiltration occurred in both tolerant and rejecting strain combinations, and that donor effector cells were reduced systemically but detected in the tolerant liver, also suggested that recipient T cells underwent cell death after interacting with the donor hepatic parenchyma itself. However, the exact cellular interactions, immune pathways, and mode of cell death were yet to be discovered. The use of transgenic mouse models deepened our understanding of early events after LT and provided further mechanistic insight into these processes.

## The Induction Phase—Knowledge Gained From More Recent Mouse Studies

### Rationale for Using Transgenic Mouse Models in LT Studies

Characterization of the molecular and cellular basis of LT tolerance requires assessing the activation, phenotype, function, and fate of alloreactive T cells in the recipient. This is challenging with regard to the polyclonal alloresponse studied in rat models, as graft-reactive T cells represent a heterogenous population recognizing often uncharacterized epitopes with varying affinities, and comprise only 1–10% of total T cells diluted within a large pool of non-alloreactive recipient T cells. Thus, while rat models have considerably advanced our knowledge of immune responses in LT, the complex polyclonal response and the limited availability of tools and reagents to analyze this response have significantly hindered progress.

Although LT in the mouse is a technical “tour de force,” only successfully achieved by a handful of surgeons worldwide, the large number of reagents as well as transgenic, knock out, knock in, and reporter mouse lines available offer unparalleled tools for analysis of the immune response that is simply not feasible in rats. TCR transgenic mice in which all CD8 or CD4 T cells express a monoclonal T cell receptor recognizing a specific alloantigen are particularly useful tools, as all T cells in the mouse recognize the same ligand with the same affinity. This response is thus monoclonal, homogenous, and thus easier to interpret. Importantly, TCR transgenic T cells can also be labeled with a cellular dye or via the expression a transgenic fluorescent reporter protein before being adoptively transferred into mice undergoing transplantation. This allows their identification and tracking in the host, and thus the assessment of cell numbers, dynamics, and fate in the recipient. Several studies have used this approach to characterize the function and fate of T cells activated by their cognate antigen in an intact liver in great detail. These studies have revealed previously unreported properties of the liver that have significant consequences for LT.

### Recognition of Cognate Antigen in the Liver by Effector and Naïve CD8 T Cells

Early studies investigating the fate of *in vitro* activated CD8 and CD4 T cells adoptively transferred into syngeneic recipient mice reported efficient intrahepatic trapping of donor CD8 T cells. As T cells retained in the liver were apoptotic, these investigators suggested that the liver was a disposal site for terminally differentiated mature CD8 T cells (21) or for the active killing of effector cells (22), and that this process was linked to tolerance in this organ (23). Although this “graveyard model” gained some traction, it failed to explain how a non-antigen dependent passive process could drive antigen-specific tolerance. Apoptosis of CD8 T cells upon secondary activation in the liver would also preclude the generation of effector or memory T cell responses, and would be difficult to reconcile with clinical observations: in particular, the effective clearance of hepatotropic pathogens such as the hepatitis A virus, which undergoes universal clearance, and the hepatitis B and C viruses, where infection resolves in 90 and 30% of individuals, respectively (24). Additionally, this model is inconsistent with the high numbers of functional effector memory T cells detected in this organ (25–27). Recent studies have provided some insight into the fate of activated T cells in the liver. *In vitro* activated CD8 T cells adoptively transferred into non-antigen expressing recipient mice survive and differentiate into liver resident memory T cells (T_RM_) (28), a recently described memory T cell subset that plays a key role in intrahepatic immunity characterized in one of our recent studies (25). Our studies suggest that the fate of adoptively transferred CD8 T cells recognizing hepatically-expressed antigen depends on the intrahepatic antigen load. While a low number of antigen-expressing hepatocytes were cleared, allowing the survival of transferred CD8 T cells, expression of cognate antigen by a high number of hepatocytes led to the silencing of these CD8 T cells by inducing death or functional exhaustion associated with high expression of PD-1 (29). This latter scenario would be the one encountered in liver transplantation.

### Fate of Naive CD8 T Cell Activated Within the Liver

Naïve alloreactive T cells continuously recirculate via blood and lymph, and most are found in lymph nodes and spleen where they transit for several hours before exiting and rejoining the circulation. This recirculation pattern allows naïve T cells to be exposed to antigen presenting cells in both lymph nodes and spleen, but also within the hepatic sinusoids [reviewed in (30)]. Although prior dogma held that activation of naïve T cells is restricted to lymphoid organs and cannot occur in extra-lymphatic tissues, the unusual interactions between the liver and activated T cells, as well as a series of early *in vitro* studies showing that hepatocytes could function effectively as antigen-presenting cells (31–33), prompted us and others to test whether this paradigm applied to the liver.

The fate of naïve CD8 T cells expressing a transgenic TCR recognizing an allo-MHC molecule or antigen expressed in the liver was investigated by several groups. While some groups focused on liver sinusoidal endothelial cells (LSECs) or stellate cells, our studies focused on hepatocytes as they are the selective target of prevalent liver pathogens, including the major human hepatitis viruses and malaria. Our early *in vitro* studies demonstrated that hepatocytes are efficient antigen presenting cells able to activate naïve CD8 T cells (31–33). However, T cells activated by hepatocytes underwent a differentiation program distinct from that triggered by dendritic cells (DCs), the major professional antigen presenting cell population: while naïve CD8 T cells activated by DCs became potent cytotoxic T cells that survived for up to 5 days in culture, naïve transgenic CD8 T cells activated by hepatocytes transiently became CTLs, but died prematurely within three days following primary activation (32) due to insufficient costimulation, and failure to express adequate levels of IL-2 and the survival gene bcl-x_L_ (33). To assess whether naïve CD8 T cells could be directly activated by hepatocytes *in vivo*, naïve TCR transgenic CD8 T cells recognizing the allo-MHC molecule H-2K^b^ were transferred into recipient mice expressing H-2K^b^ as a transgene under the control of the sheep metallothionein or mouse albumin promoters, restricting expression to hepatocytes. CD8 T cells were rapidly retained in the liver after adoptive transfer, and underwent subsequent activation and proliferation (34, 35). Retention and activation were antigen-specific, as T cells were not retained in a non-antigen expressing liver (34). Restriction of H2-K^b^ expression to hepatocytes excluded T cell activation in lymphoid tissues, suggesting that hepatocytes activated naïve CD8 T cells independently of secondary lymphoid tissues (34, 35). Electron microscopy studies provided visual evidence of these interactions, and showed that they occurred through the fenestrae of liver sinusoidal endothelial cells (36). This was the first report of primary T cell activation outside secondary lymphoid tissues. By tracking hepatocyte-activated transgenic CD8 T cells in the recipient, we demonstrated that intrahepatic activation by hepatocytes promoted antigen-specific tolerance, whereas effective immunity to hepatically expressed antigen required primary activation of CD8 T cells in the secondary lymphoid organs (35). These results highlighted the role of the site of primary activation in determining the outcome of the CD8 T cell response for the first time, a phenomenon potentially acting as a key mechanism driving tolerance after liver transplantation. The potential mechanisms determining the hepatic silencing of the CD8 T cell response will be detailed below.

By generating bone marrow irradiated chimeras in which H-2K^b^ expression was restricted to bone marrow-derived cells, we showed that bone marrow-derived cells were sufficient for intrahepatic retention of CD8 T cells (37). As Kupffer cells (KCs) are the main sinusoidal cell derived from bone marrow in radiation-induced chimeric models, these results suggest that antigen expressing KCs could also activate naïve CD8 T cells.

Liver sinusoidal endothelial cells (LSECs) and hepatic stellate cells have also been demonstrated to be capable of activating naïve CD8 T cells (38, 39). LSECs have been the subject of several studies, as they are scavenger cells able to process antigen via the direct presentation pathway for presentation in the context of MHC class I, but are also able to cross-present antigen (40), a property largely restricted to certain subsets of dendritic cells. Unlike hepatocytes, these cells express low levels of MHC class II in addition to MHC class I, and could therefore act as antigen presenting cells for CD4 T cells (41). The role of LSEC and other liver cells in presenting antigen has been the subject of several previous reviews (42–44).

Collectively, these results suggest that a variety of cell types can activate naïve CD8 T cells within the hepatic sinusoids. To our knowledge, the liver is the only non-lymphoid organ that supports primary CD8 T cell activation. The liver owes this property to its unique architecture, being comprised of a myriad of narrow sinusoids lined with perforated endothelium and harboring liver resident macrophages within their lumens. When combined, these features create a unique environment in which the blood flow is slower than in other capillary beds, allowing selectin-independent recruitment of leucocytes within the liver (30), and direct contact with a range of potential antigen presenting cells not possible in other non-lymphoid organs with continuous endothelium and higher capillary flow rates. This property may be critical for understanding immunity in this organ and tolerance after LT.

### Studies in LT Using Transgenic Mouse Models

Liver transplantation creates altered conditions for the recipient immune system that are expected to have a profound effect on T cell activation:

*1. The inflamed microenvironment related to surgery*: Inflammation associated with surgery and ischemia-reperfusion injury alters expression of molecules that regulate T cell activation (MHC, adhesion, and costimulatory molecules and cytokines) (45). These changes might promote bystander activation of non-graft reactive T cells or recruit T cells that would not contribute to a physiological response under uninflamed conditions, i.e., those recognizing low affinity ligands. To assess whether procedural associated inflammation alters intrahepatic T cell activation, we have recently developed a mouse LT model in which labeled naïve TCR transgenic CD8 T cells recognizing the allo-MHC molecule H-2K^b^ can be easily identified after adoptive transfer into recipient mice receiving syngeneic or allogeneic H-2K^b^ expressing liver grafts (46). By assessing early immune events in this model, we have shown that naïve T cells are retained and activated in the liver allograft with similar kinetics to that observed in a non-transplant setting (46). Importantly, naive alloreactive CD8 T cells were not retained or activated in syngeneic liver grafts (46), providing convincing evidence that retention of alloreactive CD8 T cells in the transplanted liver is antigen-dependent and is not altered by the surgery. These results confirm similar observations made by Kamada in the rat (7).

*2. Disruption of T cell activation in lymphoid tissues by PL:* In a physiological setting, liver antigen would be processed by two different pathways leading to presentation by different cells in distinct compartments. While antigen processing via the direct pathway of MHC class I presentation leads to presentation by hepatic cells in the liver, antigens captured and cross-presented by dendritic cells are presented in lymphoid tissues (29). Although these two pathways contribute after liver transplantation, PL migration to lymphoid tissues allows a large cohort of cells that are not specialized in antigen presentation to migrate to areas dedicated to antigen presentation normally initiated by a low number of dendritic cells. This enables a third unphysiological T cell activation pathway, which disrupts physiological antigen presentation in lymphoid tissues.

The impact of PL migration in lymphoid tissues was recently investigated in our mouse LT model (46). Our results confirmed that PL migration occurred almost as soon as the recipient blood starts flowing into the graft and continues to occur within the first hours after transplantation (46). By dissecting migration patterns of different PL cell subsets, we showed that preferential migration to recipient spleen or lymph nodes resulted in differences in PL composition between these two compartments that reflected the physiological composition of these compartments: for example, while recipient spleens contained mostly donor B cells, recipient lymph nodes contained mostly donor T cells. Although most cells migrated out of grafts, most NK T cells and a significant proportion of NK cells stayed within liver allografts. The remaining NK cells circulated via the blood and were not detected in lymphoid organs. Intrahepatic retention of NKT cells confirms their tissue residency, and is consistent with findings from parabiotic experiments (47). Most importantly, migration of PLs was initially very similar in syngeneic and allogeneic recipients; (46) however, a difference between syngeneic and allogeneic two recipients was observed after 2 days, as PLs numbers dropped in the allograft recipients, reflecting their killing by alloreactive recipient CTLs (46).

Activation of alloreactive TCR transgenic CD8 T cells in recipient lymphoid tissues was a very early event, being detected at 5 h post-transplant (46). By transplanting liver allografts ubiquitously expressing a reporter protein into recipient mice harboring labeled transgenic alloreactive CD8 T cells, we were able to visualize interactions between most alloreactive transgenic CD8 T cells and PL in lymphoid tissues as soon as 5 h after the surgery, suggesting that the observed activation of alloreactive CD8 T cells was directly initiated by PLs (46). Furthermore, all alloreactive transgenic T cells contained in lymph nodes and spleen were activated, suggesting that they were recruited at once, a result that is not entirely surprising considering the large number of PLs contained in a liver allograft.

As PL-mediated T cell activation is such a prominent immune event during the first days after liver transplantation, PLs were initially considered the main cell driving T cell activation after transplantation, and their role was examined in most early studies in rat models (48). The seminal report by Starzl et al. describing PL-mediated microchimerism (12) influenced the field and reinforced this trend. As mentioned earlier a role of PL mediated activation in tolerance is supported by our early study showing that (i) adoptive transfer of large numbers of donor splenic or liver leucocytes immediately after transplantation converted rat liver allograft rejection to long term acceptance and prolonged the survival of rat kidney allografts; (49) (ii) irradiation of rat liver allograft before transplantation promoted rejection in normally tolerant strain combinations; (16) and (iii) “parking” of irradiated livers in syngeneic hosts prior to allotransplantation reconstituted tolerance (16). These findings raise some key questions: firstly, how do PL mediate tolerance? Secondly, if PL mediate tolerance after liver transplantation, why do PL from other solid organs fail to induce tolerance after transplantation?

It has long been observed that transfusing the recipient with blood from the graft donor prior to transplantation prolongs allograft survival. This effect, first described by Medawar in 1946 (50), has become known as “the blood transfusion effect,” and it was initially suggested that tolerance induced by liver allografts resulted in similar fashion due to the high number of PLs transplanted along with this large organ. High PL numbers might create “high dose tolerance,” promoting activation-induced cell death (AICD) of alloreactive CD8 T cells (48). AICD describes cell death occurring when activated T cells re-crosslink their TCR in the presence of IL-2 (51). As they are activated, T cells co-express death molecules and their ligands on their cell surface. If T cells are in close contact with each other, they trigger the death receptor pathway of other cells, resulting in apoptosis (51). T cells are highly sensitive to AICD during the first 2–3 days after primary activation, but as they start to overexpress FLIP (FLICE inhibitory protein) between 24 and 48 h, they become resistant after 48 h (52). While AICD of CD4 T cells is mediated by FasL, AICD of CD8 T cells involves TNFRII (53). However, AICD is a phenomenon observed *in vitro*, and it remains unclear whether it occurs after transplantation *in vivo*. Nevertheless, the timeframe during which allograft reactive T cell death is observed after LT, within the first 2–3 days post-surgery, coincides with the period in which T cells have been found to be sensitive to AICD *in vitro*. Thus, early PL-mediated apoptosis of recipient alloreactive CD4 and CD8 T cells in recipient lymphoid tissues after LT might occur in association with this, or a closely related, process.

It must be noted that although PL have been demonstrated to induce tolerance or prolong graft survival in rat models, transfer of large numbers of donor-derived splenocytes or intrahepatic lymphocytes do not lead to tolerance of rat kidney and heart allografts (54), suggesting that the spontaneous acceptance of liver allografts is not solely mediated by PL.

**Figure 1:**
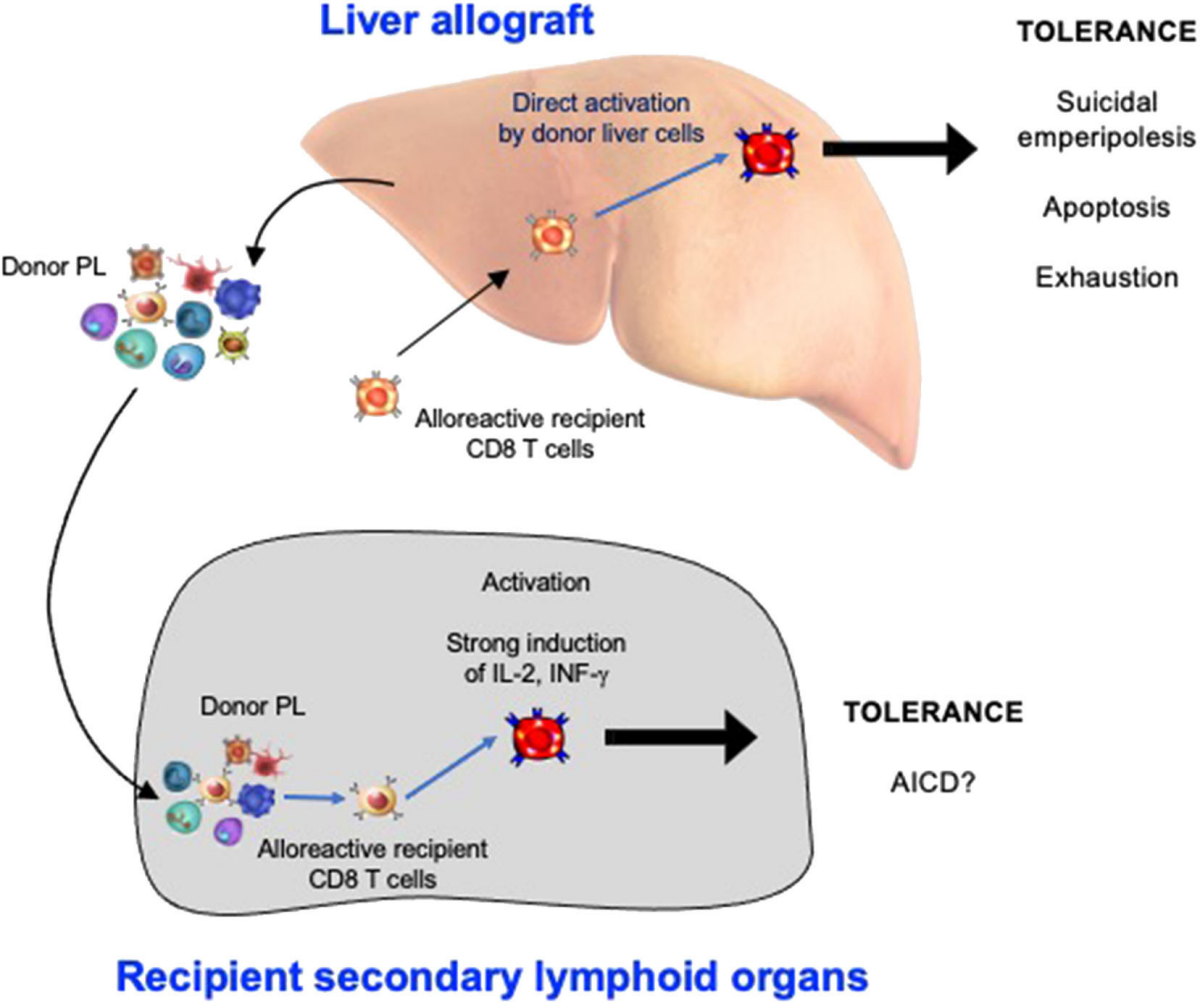
The Induction phase of liver transplant tolerance: pathways of alloreactive CD8 T cell silencing in recipient secondary lymphoid organs and within the hepatic allograft. PL, passenger leukocytes; AICD, activation induced cell death.

*3. The non-physiological expression of alloantigen by all cells of the liver allograft:* As naïve CD8 T cells can undergo primary activation in the intact liver, they might also be activated by hepatic cells in liver allografts. We confirmed that this was the case by tracking graft-reactive CD8 T cells in the recipient of a liver allograft: naïve allograft-specific CD8 T cells underwent activation in the spleen and lymph nodes, but were also concomitantly retained and activated within the liver allograft, although not within the livers of syngeneic graft recipients (46). As primary CD8 T cell activation in the livers of intact animals has been shown to promote tolerance (35), this pathway might also be a key mechanism promoting tolerance after LT. The fate of T cells activated within the liver allograft, and the relative contributions of this pathway vs. the PL-mediated activation pathway occurring in recipient lymphoid tissues, have not yet been delineated. However, studies performed in intact livers do yield some clues. To investigate a setting relevant to liver transplantation in which donor MHC molecules are expressed by all liver cells including leucocytes, donor transgenic CD8 T cells were adoptively transferred into recipient mice ubiquitously expressing their cognate antigen, the alloantigen H-2K^b^. We made the surprising finding that 80–90% of T cells undergoing primary activation within the liver were rapidly eliminated by a non-apoptotic mechanism (55). Deletion resulted from T cell invasion of hepatocytes, a process leading to their rapid destruction in LAMP-1^+^ lysosomal compartments. Cell-in-cell structures can arise by “emperipolesis” (56), a process long observed in liver sections in autoimmune hepatitis and viral hepatitis induced by HBV, HCV, and Epstein-Barr virus infections. To distinguish our findings from the classical form of emperipolesis that does not imply destruction of the invading cell, we have termed this non-apoptotic death *suicidal emperipolesis* (55). Although most liver-activated alloreactive CD8 T cells disappeared by suicidal emperipolesis, 10–20% of H-2K^b^-specific CD8 T cells survived this process. These residual cells displayed poor effector function, expressed high levels of the pro-apoptotic molecule Bim, and underwent premature cell death via apoptosis, thus limiting their ability to induce liver damage (57). Although these two processes of CD8 T cell death eliminated most donor T cells, a small population of residual H-2K^b^ T cells persisted at later time points. However, these cells expressed high levels of PD-1, and were not functional, suggesting that they were exhausted (29). Thus, our results strongly suggest that CD8 T cells activated within the liver are tolerized by at least 3 mechanisms: death by suicidal emperipolesis, Bim-mediated apoptotic cell death, and functional exhaustion. We hypothesize that similar mechanisms operate in the hepatic allograft after LT, with PL also inducing parallel activation leading to apoptosis of graft-reactive CD8 T cells within the recipient lymphoid tissues (58). This model is supported by previous findings by Qian et al. (59). suggesting that T cell deletion is the most important mechanism mediating tolerance after mouse liver transplantation. It is also consistent with reports suggesting that both the hepatic parenchyma and PLs contribute to tolerance induction after rat LT (18) and that human liver transplantation is associated with deletion of T cells bearing specific TCR beta sequences (60). Tolerance to liver allografts is consistent with our studies showing that persisting high levels of intrahepatic antigen expression, a situation akin to that associated with organ transplantation, are generally associated with tolerance (29). In contrast, low levels of intrahepatic antigen expression, for example where antigen is expressed by low numbers of hepatocytes or via transient intrahepatic antigen presentation following administration of exogenous peptide, promote functional responses, antigen clearance and T cell survival (29). The multiple pathways occurring in the early tolerance phase post-LT are summarized in Figure 1 and Table 1.

**Table 1:** Summarizing the main features of the induction phase of liver transplant tolerance.

**Time after liver transplantation**	**Events occurring in SLOs**	**Events occurring in the liver**
0–12 h	• The large bulk of PL enter SLOs within the first few hours after the surgery • PL activate alloreactive CD8 T cells located in SLOs	• Most PLs rapidly leave the liver except for NKT cells and some NK cells • Primary CD8 T cell activation by liver cells • Clearance of activated CD8 T cells by Suicidal emperipolesis?
12–24 h	• PL activated alloreactive CD8 T cells express cytokines (IL-2, IFN-γ) and start to proliferate	• CD8 T cells not cleared by suicidal emperipolesis express cytokines (IL-2, IFNγ)
24–48 h	• PL activated alloreactive CD8 T cells express cytokines (IL-2, IFN-γ) die by AICD	• CD8 T cells that were not cleared by suicidal emperipolesis fail to survive and die by neglect
After 48 h	• Some T cells survive AICD and leave SLOs	• T cells survive AICD in SLOs enter the liver but become rapidly exhausted and silenced
After 30 days		• Regulatory T cells start to be generated and maintain tolerance

## The Induction Phase—Human Studies

Only a few studies have examined the induction phase of tolerance in human liver transplant recipients. We described an increase in interferon γ producing peripheral blood mononuclear cells in patients who did not have a subsequent early episode of allograft rejection (61). This was consistent with studies in experimental animals showing tolerance was associated with an early immune activation phenotype (15). However, such studies are confounded by the relatively early introduction of immunosuppressive therapy in human transplantation which may blunt or inhibit such a phenotype, as discussed below.

One of the controversies regarding the induction of LT tolerance has been the role played by regulatory T cells (Tregs). Depletion of recipient CD25^+^CD4^+^ T cells at 100 days post liver transplantation (62) or before transplantation (63) using anti-CD25 mAb induced acute liver allograft rejection suggesting that CD25^+^ CD4^+^ Tregs were critical in maintaining tolerance. Some studies have shown that CD8 T cells expressing CD103 and Foxp3 with regulatory function were increased in recipients spontaneously accepting liver grafts suggesting that they might also contribute to the induction of tolerance (64). Kamada et al. described two phases of *in vitro* immunosuppressive activity of splenocytes harvested from tolerant rat recipients of LT, with splenocytes harvested from days 5–28 and from >20 weeks able to suppress mixed lymphocyte reactions, however those obtained during the intervening period lacked such activity (65). More recent studies have demonstrated that splenocytes from long term tolerant animals were able to induce liver transplant tolerance, however transfer of splenocytes harvested at 30 days did not (8). Thus, the appearance of early Tregs and any role that they may play in the induction of spontaneous tolerance has been under significant scrutiny.

An important role for early Treg induction in liver tolerance has been suggested by the success of a novel protocol used by Todo and colleagues in human live donation LT (66, 67). This protocol involved the generation of donor Tregs *in vitro*, followed by their transfer to the recipient at day 13 post-transplant. Successful withdrawal of immunosuppression in 7 of 10 patients without any rejection suggested that tolerance had indeed been induced (operational tolerance). However, whether such cells were acting to induce tolerance during the early induction phase or at later time points cannot be ascertained. Furthermore, it is unclear whether immunological events following transfer of *in vitro* generated Tregs mirror those developing in the early phase post-LT in the absence of administration of such cellular therapy.

It should also be pointed out that in humans immune-ablative induction protocols followed by early cessation of immunosuppression have not been successful in tolerance induction. Indeed, in one study a significant increase in allograft rejection was seen and the study was prematurely terminated (68). Such results support the concept that some form of immune activation is also required for human liver tolerance, and that protocols that allow for this may be necessary to manipulate the balance of tolerance/rejection at an early stage.

### The Induction Phase—Comparison With the Maintenance Phase in Animals

Although there is no evidence that Tregs can induce LT tolerance, it has been well demonstrated in animal models that once tolerance is established Treg cells can transfer tolerance and prevent rejection (45). This usually takes about 70–100 days post LT to uniformly occur.

### The Induction Phase—Comparison With the Maintenance Phase in Humans

As mentioned previously, the induction phase in humans has been sparsely investigated, and studies have been complicated by early immunosuppression used extensively in human transplantation. In contrast, several studies of functional tolerance, or so called “operational tolerance,” have been undertaken. This is defined as a subgroup of patients who, upon withdrawal of immunosuppression, do not reject their liver allografts. It is rather uncommon if the frequency is defined from the time of transplant itself, occurring in probably around 5% of patients. However, if patients are carefully selected by criteria including long duration post-transplant, already on minimal immunosuppression, pediatric recipients, and no autoimmune disease then between 20 and 40% of long-term LT patients can be successfully withdrawn from immunosuppression (69). More recently, the use of liver biopsy, in particular the finding of normal histology (70) and absence of donor specific antibodies are also thought to be important predictors, although the later factor remains less well delineated (71).

Patients under study for predictors of operational tolerance have displayed various molecular and cellular signals associated with peripheral blood leucocytes and with the liver itself. Predictive biomarkers of liver transplant tolerance associated operational tolerance with an increase in peripheral blood Tregs, NK cells, or γδ T cells (72) as well as genes expressed by these cell types such as sentrin-specific peptidase 6 (SENP6) and Fem-1 homolog C (FEM1C) (73, 74). In one study an increase in gene expression associated with iron metabolism was seen in the liver (75). However, the findings of such studies have been inconsistent, and there is currently an immunosuppression withdrawal trial underway using one particular molecular marker subset as a starting point for withdrawal (76). It is likely that Treg cells will be important for successful withdrawal of immunosuppression in this phase, although this still remains to be defined. Some data suggest that long term use of mTOR inhibitors may favor the emergence of Tregs thus potentially promoting the maintenance phase of tolerance (77). The exact details of these approaches are outlined in other article(s) of in this edition.

## Conclusion

Experimentally, it is clear that the induction phase post-LT is associated with, and probably causative of, LT tolerance via immune activation events. If the same applies in humans, then current practices of early use of high dose immunosuppression in clinical LT may inhibit the induction of such early immune activation processes and thus be detrimental to tolerance induction. This concern is supported by the failure of at least one human trial of early immunosuppression withdrawal after ablative immune induction.

In conclusion, animal models have enabled us to understand the induction phase of liver tolerance, whilst new studies in humans have revealed significant insights into the maintenance phase. The challenge is to understand how these are linked, so that we may identify potential tolerant patients much earlier in their post-transplant course and modify immunosuppression accordingly. This would have the maximum benefit of decreasing immunosuppression related comorbidities, rather than waiting for many years, after which such co morbidities may not be reversible.

## Author Contributions

All authors listed have made a substantial, direct and intellectual contribution to the work, and approved it for publication.

## Conflict of Interest

The authors declare that the research was conducted in the absence of any commercial or financial relationships that could be construed as a potential conflict of interest.
